# Editorial: Mineral solubilizing microorganisms (MSM) and their applications in nutrient bioavailability, bioweathering and bioremediation, volume II

**DOI:** 10.3389/fmicb.2023.1345161

**Published:** 2024-01-04

**Authors:** Muhammad Zahid Mumtaz, Maqshoof Ahmad, Hassan Etesami, Adnan Mustafa

**Affiliations:** ^1^Institute of Molecular Biology and Biotechnology, The University of Lahore, Lahore, Pakistan; ^2^College of Agronomy, Gansu Agricultural University, Lanzhou, China; ^3^Department of Soil Science, The Islamia University of Bahawalpur, Bahawalpur, Pakistan; ^4^College of Agriculture and Natural Resources, University of Tehran, Karaj, Iran; ^5^Key Laboratory of Vegetation Restoration and Management, South China Botanical Garden, Chinese Academy of Sciences, Guangzhou, China

**Keywords:** mineral solubilizing microorganisms, phosphate solubilizing microorganisms, nutrient bioavailability, bioweathering, bioremediation, mineral-microbe interactions, recovery of metals

Soil microorganisms are involved in mineral weathering, nutrient bioavailability, and bioremediation in the soil ecosystem. In this regard, microbes' mineral interactions can assist exogenic biogeochemical reactions. Various microorganisms cause weathering and rock decomposition through biocorrosion, bioerosion, and bioperforation by inhabiting rock surfaces or crevices (Bin et al., 2008). Microbes acquire nutrients from rock surfaces by building complex organic ligands and encouraging the release of mineral elements. Bioperforation in rock surface by microbial colonization promotes the exposed surface area of rock and intensifies its weathering by mechanical erosion, microbial destruction, and deterioration of rock particle cementation structure (Ehrlich, 1998). Microorganisms also boost mineral weathering through acidification by producing organic acids, microbial water retention, and release of CO_2_ due to microbial respiration (Chen et al., 2000). Microorganisms enhance mineral availability from geological resources through mineral breakdown and rock deterioration. Furthermore, microbial technology exploits low-grade mineral resources available for plant uptake, accelerating soil formation (Kumar et al., 2018).

Microorganisms promote soil physicochemical characteristics and nutrient cycling in soil solution (Li et al., 2006). Roots exudates facilitate mineral solubilizing microorganisms (MSM) to perform their activity. In response to the plant's nutritional needs, exudation rates increase with root surface area (Aoki et al., 2012). In addition, plants emit sugars, amino acids, enzymes, fatty acids, sterols, growth factors, vitamins, and secondary metabolites. They transform the rhizosphere into a nutrient-rich environment that may support a diverse microbial population (Vives-Peris et al., 2020). The MSM in the rhizospheric area promotes a more intense mineral bioavailability than bulk soil. Microorganisms solubilize insoluble minerals through various mechanisms, including direct dissolution, redox reactions, and enzymatic production of various acid phosphatases and phytases (Kumar et al., 2013). They solubilize minerals from the soil directly through microbial weathering and promote plant growth by improving root development, modulating phytohormones, producing siderophores and hydrogen cyanide, increasing plant enzymatic activities, controlling phytopathogen under normal as well as stress conditions (Mumtaz et al., 2017; Etesami and Adl, 2020; Saeed et al., 2021).

Industrialization in the current era negatively affects soil and crop productivity by accumulating vast amounts of minerals, including heavy metals. These heavy metals are cytotoxic even at low concentrations and cause human cancer. Their toxicity causes higher production of reactive oxygen species that reduce the antioxidant systems and affect an organism's normal functioning, ultimately leading to cell death (Tarfeen et al., 2022). The MSM can perform bioremediation of contaminated agricultural soil. These microorganisms perform bioremediation of metal minerals through biosorption to cell walls, precipitation, capturing in extracellular capsules, efflux outside the cell, transformation of metal minerals to a less toxic state, and ligation inside cell leading to adsorption of metal minerals ions (Riseh et al., 2022). Bioremediation of metal minerals depends on environmental factors (temperature, pH, electron acceptor, nutrients, etc.), contaminant properties, biodegradative genes in organisms, and bioavailability of contaminants to degrading microorganisms within the site (Maier, 2000). In the current Research Topic, metal bioavailability was considered to be the most crucial factor in bioremediation. The bioavailability of minerals also has a vital role in rocks and minerals weathering and nutrient availability for crop growth and productivity. The MSM have biologically activated metabolic systems that are eco-friendly and cost-effective and have various mechanisms for weathering, bioavailability, and bioremediation of minerals.

This Research Topic aims to uncover the role of MSM in mineral weathering, nutrient availability, and bioremediation. This topic presents two reviews and four original research papers by 39 authors from seven countries that span the research on mineral-solubilizing microorganisms. This topic publishes one article on weathering, three articles on nutrient bioavailability and plant growth promotion, and two articles on the bioremediation or recovery of metal minerals. These articles give insight into ongoing issues and provide a basis for further study on the application of MSM in nutrient availability and bioremediation. Here, we summarized some highlights from the six articles published on this Research Topic.

## Mineral bioweathering through mineral solubilizing microorganisms

Microbial diversity is a crucial factor for soil formation from rock weathering. Chen et al. reported a higher abundance of fungi over bacteria during premature colonization on limestone. Fungal communities were remarkably unaffected by nutrient solutions, organic acid, inorganic acid, and microbial competition, while bacterial communities were robust and constant. They observed the dominant fungal phyla were Ascomycota, Basidiomycota, and Chytridiomycota, while the dominant bacterial phyla were Proteobacteria, Bacteroidota, and Actinobacteriota, which have their application in weathering of limestone.

## Nutrient bioavailability through mineral solubilizing microorganisms

The MSM are associated with plants to promote essential mineral uptake. Suraby et al. reported the solubilization of insoluble phosphate compounds, including AlPO_4_, FePO_4_·4H_2_O, Ca_3_(PO_4_)_2_, and hydroxyapatite by fungal strain *Penicillium olsonii* TLL1. This strain promoted Arabidopsis, Bok choy, and rice growth in vermiculite soil under phosphorus-limiting conditions. Rizwanuddin et al. emphasized the solubilization of soil organic phytate by phytase-producing microorganisms. MSM produces phytase enzymes that enhance the phytate mineralization through the cleavage of phytate ester bonds. Transgenic plants are generated by inserting microbial phytase-producing genes to promote phytate mineralization and phosphorus availability in plants. Raimi et al. reported that organic fertilizers support highly functional and more robust interactions within the rhizosphere bacterial community. Organic fertilizers demonstrated functionally versatile bacterial community structures characterized by plant growth-promoting genera such as Agromyces, Bacillus, and Nocardioides over traditional fertilizers. Rhizospheric bacterial communities had higher diversity richness and unique bacterial genera in organic farms than in conventional farms.

## Bioremediation through mineral solubilizing microorganisms

Metal minerals at high doses are recalcitrant contaminants that can harm living organisms. Singh et al. reported the bioaccumulation/removal of rare earth elements from hazardous industrial waste of compact fluorescent lamp acid extract using synchronous culture of extremophilic red algae *Galdieria sulphuraria*. The algal efficiency to accumulate rare earth elements was augmented by adding 6-benzylaminopurine and 1-naphthaleneacetic acid, which could be the solution for the removal or recycling of wastes containing rare earth elements. Biswal and Balasubramanian reviewed the performance of various microbial agents to extract cobalt and lithium from the solid matrix of spent lithium-ion batteries. Bioleaching of dissolved metal from spent lithium-ion batteries is effective through bacterial strains, including *Acidithiobacillus ferrooxidans* and *Acidithiobacillus thiooxidans*, and the fungal strain *Aspergillus niger*. The production of sulfuric acid from bacterial strains and citric acid, gluconic acid, and oxalic acid from fungal strains are the dominant metabolites involved in cobalt and lithium bioleaching.

## Author contributions

MM: Conceptualization, Data curation, Formal analysis, Investigation, Methodology, Project administration, Supervision, Validation, Writing—original draft, Writing—review & editing. MA: Formal analysis, Supervision, Validation, Writing—review & editing. HE: Formal analysis, Validation, Writing—review & editing. AM: Data curation, Formal analysis, Writing—review & editing.
